# Healthcare workers’ views on type 2 diabetes mellitus management at selected clinics in Mthatha

**DOI:** 10.4102/phcfm.v16i1.4382

**Published:** 2024-05-15

**Authors:** Michael O. Ameh, Ramprakash Kaswa, Busisiwe Cawe

**Affiliations:** 1 Department of Family Medicine and Rural Health, Faculty of Health Sciences, Walter Sisulu University, Mthatha, South Africa

**Keywords:** diabetes, views, healthcare workers, management, primary healthcare

## Abstract

**Background:**

Diabetes is a non-communicable disease of global public health importance. Healthcare workers play a vital role in the management of this disease.

**Aim:**

This study aimed to explore healthcare workers’ views on managing patients with type 2 diabetes at primary health care facilities.

**Setting:**

The study was conducted at two primary health care facilities in Mthatha, South Africa.

**Methods:**

This exploratory descriptive qualitative study included 28 primary health care workers. Data were collected through individual interviews and focus group discussions and analysed using a thematic analysis approach.

**Results:**

Study participants’ views of poor control of type 2 diabetes mellitus were categorised under patient- and healthcare system-related factors. The patient-related factors included poor adherence to an ideal diabetic diet, poor medication adherence, a lack of personal glucometers, and dearth of support systems. The healthcare system-related factors identified were inadequate patient education, long waiting times at the health facilities, high patient volumes, limited resources, and delayed service provision. Proposed solutions to address poor control of diabetes included improving patient health education, providing diabetic patients with glucometers, multi-stakeholder management of diabetes, allocating designated areas for patients with chronic illnesses, improved resource allocation, and regular staff training.

**Conclusion:**

Study participants perceived an improved level of control of diabetes among patients managed at the Community Health Centres. When designing interventions for the management of diabetes, both patient and healthcare system-related factors and the proposed solutions should be considered.

**Contribution:**

This study’s findings could promote better management of diabetes at the primary health care level.

## Introduction

Diabetes mellitus (DM) is the fourth most common non-communicable disease worldwide, accounting for 32.4 million of the 56.9 million deaths in 2016.^1^ About one in eight adults, worldwide, will be living with the disease by 2045.^2^ Diabetes is characterised by chronic hyperglycaemia, which, in the case of type 2 diabetes mellitus (T2DM), could result from predominant insulin resistance with a relative insulin deficiency, to a largely secretory defect with insulin resistance.^3^ Type 2 diabetes mellitus is associated with increased mortality rates, complications, disability, and reduced quality of life.^2,4^ Comorbidities associated with diabetes, along with its complications, increase the utilisation of healthcare services and increase health expenditures for the individual, families, and society.^4,5,6,7,8,9^

Diabetes is prevalent in South Africa because of changing population demographics, urbanisation, and unhealthy lifestyle factors.^10^ The OR Tambo District Municipality, a rural setting, had the highest number of new diabetic patients in the Eastern Cape province.^11^ This finding is unusual, because it is typically individuals residing in urban settings who are usually exposed to risk factors for diabetes, such as sedentary lifestyle and consumption of foods linked with obesity. Adeniyi et al. also reported a high proportion of overweight and obese adults because of changing dietary patterns in this region.^12^

The care of the majority of diabetic patients takes place at the primary health care level, which is the entry point for the healthcare needs of the community.^13^ The control of diabetes has been reported to be poor in several regions of the world, including the region where this study was conducted.^14,15,16,17,18,19^ For most patients, control of diabetes is said to be poor when the glycosylated haemoglobin is greater than 7% or the fasting blood glucose is greater than 7 mmol/L.^20^

Previous studies in South Africa evaluated the monitoring and management of patients with T2DM in an under-resourced healthcare setting and diabetic patients’ perspectives on the challenges of glycaemic control. It was found that poor control of diabetes was because of clinical inertia, which includes inadequate healthcare worker interventions, poor patient participation in their own care, and ineffectiveness of the healthcare system.^21,22^

It is essential to understand the dynamics of poor glycaemic control in people with diabetes, as treatment outcomes are influenced by the quality of care, healthcare workers’ roles, patient behaviour, and organisational performance. Healthcare workers play a vital role in implementing the processes of care of patients living with diabetes,^21^ but their views on management of the disease at the primary healthcare level have been understudied. We, therefore, set out to explore the views of healthcare workers on managing patients with T2DM at primary healthcare facilities. The primary objective of the study was to explore the healthcare workers’ perception of factors associated with the control of diabetes among patients managed in primary care. The secondary objective was to explore the interventions suggested by healthcare workers for the control of diabetes in primary care.

## Research methods and design

### Study design

This study adopted an exploratory descriptive qualitative design in exploring healthcare workers’ views on managing individuals with T2DM at the selected Community Health Centres (CHCs).

### Study setting

The King Sabata Dalindyebo (KSD) Local Municipality is the largest of the five local municipalities that form O.R. Tambo District Municipality in the Eastern Cape province of South Africa. It is a rural local municipality with a population of about 520 000 persons and has high rates of dependence on social grants because of high rates of unemployment. Over 99% of the population is described as black African, of which over 85% speak Isi-Xhosa as their first language^23,24^; other local languages include English and Afrikaans. Majority of the population resides in rural areas; the two largest urban centres are Mthatha and Mqanduli.^23^

The public health care services in the KSD health sub-district are provided by 1 central hospital, 1 regional hospital, 1 district hospital, 5 CHCs, and 42 clinics. The catchment areas of three of these CHCs include Mthatha.

For this study, these CHCs were divided into two strata, based on geographic location and the catchment areas of the population. We selected one CHC from each stratum. Ngangelizwe CHC, which serves about 8000 persons per month according to the KSD health Sub-district office (unpublished data), was chosen because it is the largest CHC servicing a township community within Mthatha. Also, Mbekweni CHC, servicing about 3500 persons per month, was selected as it is the largest rural CHC servicing an area located about 20 km away from Mthatha.

At the CHCs, the nurses screen individuals for diabetes while the doctors diagnose patients with the disease, screen for complications, initiate patients on medication, monitor for adverse events from medication, and do routine follow-up of patients with the disease, including annual eye and feet examinations in accordance with the Society for Endocrinology, Metabolism and Diabetes of South Africa (SEMDSA) recommendations.^20^ Individuals with complications from diabetes are referred to higher levels of care.

### Study population, sample size and sampling

The study population comprised of professional nurses, doctors, and pharmacists currently providing direct clinical care to all patients, including adult diabetic patients, at the selected CHCs. Inclusion criteria included those who had provided direct clinical care to adult diabetic patients for at least 6 months before the conduct of the study and were willing to participate voluntarily. Exclusion criteria included those who were not involved in clinical care when the study was conducted. Interim analysis was conducted after every five individual interviews to determine the point of data saturation by the research assistant and the principal researcher. The eligible participants were identified at each CHC by the facility manager and purposively selected to ensure a range of health professionals.

To gain better understanding of the healthcare workers’ multidisciplinary care of individuals with T2DM, two focus group discussions (FGDs), comprising a group of doctors, nurses, and pharmacists (six participants from the initial individual interviews) were also held at each study site.

### Data collection

An interview guide was developed by MOA (the principal researcher) based on the recommendations in the SEMDSA guidelines.^20^ A pilot of the interview guide was carried out on two participants at each study site, to evaluate the content, relevance, and appropriateness of the questions to the research objectives. All the authors were satisfied with the questions as supported by the feedback from the participants in the pilot study. However, pilot data were not included in the final study, which took place between October 2020 and December 2020.

The interview guide was organised into four sections, which include relevant demographic data, overview of knowledge of diabetes, status and factors influencing glycaemic control, and recommendations on how to improve glycaemic control among patients living with diabetes. In addition to these broad topic areas, the interviewer was trained to probe for additional information emanating from the participants. Relevant demographic data (age, sex, professional category, and years of experience) of participants were obtained through interview. Participants were asked the following questions: What are your views about diabetes as a disease and its management? What do you think about the control of diabetes in your patient population? What factors are affecting diabetes control in your practice? What more can be done to improve glycaemic control in your patients?

All interviews were conducted on site at the CHCs, during lunchtime, and with social distancing protocols and other measures in place to minimise the spread of coronavirus disease 2019 (COVID-19).

Individual interviews and FGDs were conducted by a research assistant who is experienced in conducting qualitative interviews and was trained for this study using the interview guide. The interviewer explored the key topics using open-ended questions to elicit in-depth information from the participants. In addition, the interviewer probed for important information when this did not arise spontaneously. Each individual interview lasted about 25 min in a quiet office while ensuring the participant’s privacy.

The interviews were audiotaped, and the interviewer also kept field notes of the process. The interviews were conducted in English, which was acceptable to all the participants.

The key topic areas were presented for discussion within the focus groups. The interviewer facilitated a 30 min FGD at each study site using English as the language of communication. The verbal responses were audiotaped, while the non-verbal cues were directly observed and documented by the interviewer. All the participants were encouraged to participate actively in the discussions. The FGDs were carried out in each facility’s boardroom, which allowed for social distancing.

### Data analysis

Audiotaped data from the individual interviews and FGDs were transcribed verbatim, in English, with the aid of *Otter,* a commercial transcribing service (https://otter.ai). To ensure the accuracy of transcripts, the principal researcher and R.K. independently cross-checked excerpts of the transcripts with the recordings and notes. Field notes were reviewed by the principal researcher for additional information.

Data were analysed using a thematic analysis approach.^25^ Transcripts were analysed with the NVivo (Lumivero, Denver, Colorado, United States of America) version 12 software for qualitative research in the following steps:

- Firstly, the principal researcher and the research assistant familiarised themselves with the data by reading through the transcripts several times and actively observing patterns and meanings in the data.
- Secondly, initial codes were created to represent meanings and patterns observed in the data.
- Thirdly, verification of the assigned codes was conducted independently by B.C. and R.K., who further identified interesting excerpts and coded appropriately.
- Fourthly data from similar codes were collated together and organised to themes and sub-themes by the principal researcher.
- Lastly, the emerging themes and sub-themes were subsequently reviewed by B.C. and R.K.

### Trustworthiness

Triangulation of both data sources (doctors, nurses, and pharmacists) and methods (individual interviews and FGDs) as well as piloting of the interview guide help to improve the credibility of the findings. Detailed description of the study procedure, availability of audiotaped records, field notes and transparency of the research process help to improve the dependability of the findings. Independent assessments of the audiotaped recording and transcripts as well as coding of emerging patterns and meanings help to improve the confirmability. Findings were also corroborated by verbatim quotes of the participants. Adequate description of the study setting and the study participants elucidate on the transferability of the findings.

The principal researcher was a final year registrar in family medicine based at the Mthatha regional hospital, and received referrals from, and gave consultative advice to clinics in the drainage area of this hospital. However, he had no informal relationship with the study participants. He also had the preconception that diabetes was not well managed at the primary health care centres (PHCs), owing to his prior observation that several of the clinical notes from the PHCs, revealed poor documentation of processes of care and outcomes in management of diabetes. Having borne these facts in mind, neutrality was maintained throughout the research process.

### Ethical considerations

The study was approved by the Faculty of Health Sciences Research Ethics and Biosafety Committee of Walter Sisulu University (Ref number 054/2020). Permission was also granted by the Eastern Cape Provincial Department of Health (Ref number Ec_202010_004) and the Sub-district Manager of KSD Health Sub-district. Finally, the researcher obtained permission to conduct the study from the managers of both CHCs where the study was conducted.

## Results

The profile of the study participants is detailed in Table 1. Twelve participants took part in the focus group interviews, six at each CHC. All focus group discussants were females. The key themes that emerged during data analysis and the corresponding exploration domains are presented in Table 2.

**TABLE 1:** Information about the participants.

Variable	Description	Number of participants	Range	Mean
Study site	Ngangelizwe CHC	17	-	-
	Mbekweni CHC	11	-	-
	Total	28	-	-
Sex	Male	9	-	-
	Female	19	-	-
Professional category	Professional nurse	23	-	-
	Medical doctor (general practitioner)	3	-	-
	Pharmacist	2	-	-
Age of participants (years)	-	-	26–59	43.3
Years of experience of participants	-	-	2–33	13.6

CHC, Community Health Centres.

**TABLE 2:** A summary of qualitative data.

Themes	Sub-themes
Healthcare workers’ perception of diabetes management	Profile of patients with diabetes. Availability of diabetes management guidelines at the primary health care centre (PHCC) level. Perceptions regarding the level of control of diabetes.
Factors affecting diabetes control	**Patient-related factors** Poor adherence to the ideal diabetic diet. Non-commitment to clinic appointment and low adherence to treatment. A lack of personal testing equipment (glucometers). A lack of support systems. **Healthcare system-related factors** Inadequate patient education. Long waiting times. High patient volumes. Limited resources. Delayed service provision (long laboratory turnaround times).
Suggestions for improved diabetes control	Improved health education of patients. Provision of glucometers to patients. Multi-stakeholder approach to diabetes management. Allocation of designated areas and clinicians for patients with chronic illnesses. Improved resource allocation. Regular training of healthcare workers on the management of diabetes.

In presenting the study’s findings, the researchers de-identified all participants and created codes for the study sites and the study participants’ professional categories. The first letter of the code identifies the study site: N for Ngangelizwe CHC and M for Mbekweni CHC. The next letter represents the professional category: N for nursing professional, PH for pharmacist, and GP for general practitioner, while the first digits were numbers assigned to each participant. Therefore, a participant coded NN1 is a nurse at Ngangelizwe, numbered one. Following the code for each participant is the gender, age, and years of work experience, respectively. The codes given to the focus group participants were FGP irrespective of the study site.

### Healthcare workers’ perceptions of diabetes management

The individuals with diabetes that were seen at the CHCs were profiled as being from the rural areas, overweight, unemployed, and had other chronic medical conditions such as hypertension:

‘The clients we are dealing with are mostly unemployed in the rural areas.’ (MN5, female, 59, 25)

‘In the past 2–3 years, I’ve watched diabetes cases increase in this facility, especially type 2 diabetes. These patients are mostly a bit overweight which also contributes to their condition. Some of the patients have poorly controlled diabetes, and most of them have controlled diabetes. Most of these patients have co-existing conditions like hypertension and other conditions.’ (MPH9, female, 34, 10)

The availability of standard guidelines for managing patients with diabetes, such as the Essential Drugs List (EDL), as well as having access to the Internet for information on managing patients with diabetes using applications (apps) such as the EDL app, was highlighted as beneficial to the treatment of patients with diabetes at the CHCs:

‘We’ve got the policies that are done by the Department of Health. Those stipulating how to do foot care of the clients and all those things.’ (NN3, female, 54, 25)

‘We are lucky here at Mbekweni health centre we have internet access; we have a Wi-Fi hotspot …, so we use that access if ever needed. But we also have the EDL app, which is online, and then we would just like to use it as a guideline.’ (MGP6, female, 32, 4)

The healthcare workers perceived improvements to the control of diabetes at the CHCs because of their observation that they had not encountered any patients with diabetic complications such as foot ulcers:

‘I feel good now because I have not yet been exposed to complications. I have not had a case where we had a patient who had suffered a stroke due to our poor management for the past year. I have not had the case of a patient who has lost a foot in amputation due to poor management or a poor wound due to our poor management of diabetes or poor adherence to their treatment, so far so good in terms of diabetes.’ (NN12, male, 28, 4)

The focus group discussants observed that the success they perceived with the control of diabetes can be attributed to the services of the medical doctors and professional nurses at the PHCs. These additions to the PHC team have enhanced the identification of impending signs of complications and prompted early interventions:

‘We have got doctors in the facility, so they are able to see patients, assess them except for the fact that they are here only during working hours, so after working hours there is no doctor, but should you have a diabetic emergency, there is a doctor in the facility for you to access. We have professional nurses, not diabetic trained but they are specialists in clinical assessment and treatment, so they are able to diagnose and treat emergencies. In terms of resources, also we have treatment for the patient that is diagnosed, and then there is a leaflet that is given to new diabetic to study at home at their comfort so that they know what foods to eat and what foods to avoid, all that.’ (FGP)

### Factors affecting poor diabetes control

The study revealed that poor adherence to the ideal diabetic diet is a limiting factor to achieve good control of the disease. The study participants attributed the poor dietary adherence to the disadvantaged financial background of the CHCs’ catchment population:

‘Then there’s the other issues then become issues of diet at home. Because most people are forced to have salt, sugar water, and bread every morning. So that is the only thing that they must eat. So, they are just people who are disadvantaged in that way, that they only have certain things that they can take, and they don’t have a choice but to take those things.’ (NGP7, male, 34, 5)

Another identified factor is that many diabetic patients miss their clinic appointments and default on their treatment because of several reasons. The reasons include being financially disadvantaged and relying on social grants to access healthcare, the fear of contracting COVID-19, lengthy distances to the clinics, and the fear of needles:

‘I think the factors that might contribute are economical, finances; some patients are coming from far areas. So, they might not be able to come on time on the due date since some they have to wait until they get paid these social support grants.’ (NGP6, female, 44, 8)

‘We have noticed during this time of lockdown in Covid one of the reasons was because the elderly were scared to come to the clinic because there were scared to contract the coronavirus and when they do come, there may be no drugs.’ (NN2, male, 39, 10)

‘Also, an injectable on our people are not the things that they are used to. Because mostly if the clients needed to be given injectables, they will default because especially the men. Because they are afraid of the needle.’ (NN3, male, 54, 25)

Many diabetic patients could not afford to purchase the personal glucometers needed to monitor their blood sugar levels because of financial constraints:

‘Financial constraints do affect the management of these diabetic patients because if we had enough budget, we would buy machines for each and every patient that is diabetic and teach them how to use them and encourage them to check their blood sugar levels every day.’ (MPH9, female, 34, 10)

The lack of support systems was identified by the study participants as a stressor to the diabetic patients, which contributed to poor glycaemic control. Community health workers were no longer available to visit and assist the patients in their homes to take their medication:

‘Stress is also another problem that patients deal with, most of our patients are elderly and do not have a stable support system, and this causes stress, and that has a major impact on controlling diabetes. Lack of village community health workers who use(d) to directly observe the patients and make it a point that they take their treatment is another point.’ (MN7, female, 56, 30)

The healthcare system-related factors are those concerned with health education of patients and availability of manpower. Education provided to diabetic patients and the services they received were insufficient to make them accept their condition and adhere to the necessary lifestyle changes. The long waiting times because of administrative proceedings during monthly and review visits at the clinics made it difficult for the healthcare workers to spend adequate time educating the patient after diagnosis:

‘Most of the time, it’s just not getting enough counselling to be able to accept your condition. Some are not counselled, they are just told you have diabetes, then they must start treatment because there is always a long queue. So, when you are told that you have diabetes, then we trying to finish the queue, then we put you on treatment today. So, there’s no time really to talk to the patient and counsel the patient.’ (NGP7, male, 34, 5)

‘Waiting time is a big problem because we have patients that sometimes collapse in the waiting room because glucose is too low. Here we normally read the reports in the morning, patients are waiting at that time in the reception; there are many places they go to before they arrive at the professional nurse.’ (NN12, male, 28, 4)

Another theme that emerged is the high number of patients who visited the CHCs. The study participants attributed this to the high numbers of patients from outside the catchment areas attending the selected healthcare facilities:

‘We have an influx of patients from everywhere, some we manage patients that are not in our geographical catchment area. They believe in our clinic, consequently, though patients become more in the waiting area.’ (NN12, male, 28, 4)

The lack of human and material resources required for optimal management of diabetes, such as optimal numbers of healthcare workers, adequate equipment in good working order, availability of a diabetic-trained nurse, availability of patient educational materials, and adequate physical space at the CHCs, was also highlighted by the study participants as contributing to the poor control of diabetes in patients managed at the PHC level:

‘We have a shortage of staff. We experience pressure due to that and at times shortage of equipment, where a client comes, and there won’t be Acutest strips, or they have expired, or glucometers won’t be having batteries.’ (NN8, female, 47, 21)

‘Lack of proper staff that is trained on the chronic, as in diabetes, we do not have any in the facility. And they are few if there are any, around the Eastern Cape as well. And the lack of equipment like glucometers, information charts, we do not have such things. And we don’t even have space where we can maybe stabilise or normalise patients with diabetes that maybe come with low blood glucose or high blood glucose.’ (NN10, female, 29, 3)

‘Certain times, for example, today, I’m not going lie, we don’t have Metformin at our stores. So, if the client is not going to take or have his or her Metformin which means that the client is not going to be able to take his medication and that could lead to poor control of diabetes.’ (NN16, male, 38, 5)

Finally, concerning healthcare system-related factors, the participants drew attention to the issue of systemic delays in the provision of other services such as laboratory services, and issues with the supply chain processes:

‘Always in Eastern Cape, we are always far behind. … [*B*]ecause we are still using here, our laboratories, they have got to take some time to come with the results and sometimes we do not have solutions to put in all the patients. So, it delays you to the quicker recovery of the clients.’ (NN3, female, 54, 25)

‘The out-of-stock medication thing is really a burden; it’s a heavy load for us. At the medical stores, there are people working there when you say, I need Metformin, to them, it does not really speak anything. It is not there, but I am here, I can see. I mean, am in desperate need of insulin, you know. So, the biggest problem here is the medication supply from suppliers.’ (NPH5, female, 40, 5)

### Suggestions for improved diabetes control

The study participants recommended involving patients through continuous education, while encouraging the engagement of multiple stakeholders. They also believed that the availability of physical and human resources at the individual and facility levels could improve the level of control of diabetes. Family support and improving staff knowledge about diabetes could also assist in achieving optimal diabetes control.

The study participants believed that involving patients in diabetes management through education and counselling can empower them to manage their condition. The focus group also believed that increasing demand for diabetic services and awareness campaigns would encourage patients to come to the clinic and improve self-care practices. Additionally, the study participants suggested that government should invest in providing glucometers and strips to diabetic patients to enhance their autonomy.

The study participants believed that involving patients through education and counselling could empower them to manage their condition:

‘Education is powerful because it empowers the patient to understand the condition, understand the management. Through education, you can stress the importance of dietary management, stress the importance of compliance with treatment. When you make someone aware of what is expected, how you are treating them, why you are treating them the way you are doing, you are involving them in their management. They become active.’ (NN12, female, 28, 4)

The healthcare workers suggested that there should be shared responsibility between the healthcare professionals and the patients. The professionals should provide adequate education and counselling to the patients, and the patients should endeavour to put the lessons into practice:

‘Adherence counselling, they must take their treatment. So, if we are increasing something like if they are taking a tablet two times, now it is not in control, and we want to change it to three times we must try to tell them so that right away it can be taken three times. And then even make them repeat them or make the sisters communicate it.’ (MGP6, male, 32, 4)

The focus group also added that increasing demand for diabetic services and awareness campaigns would encourage patients to come to the clinic and improve self-care practices:

‘There should be increased demand on the services that a diabetes patient can get in the clinic so that the patients became eager to come to the clinic. Another thing would be to have these messages be pop-up messages, even on the mobile phone even on the internet. So, I think that could also help people.’ (FGP)

Providing glucometers and strips to patients actively engages them in the care plan and could enhance their autonomy regarding managing their condition, according to the study participants:

‘The patients here only come once a month. Then only once a month, we are going to check the sugar because they do not have the machine at home. So, the government should invest maybe on providing those who’ve been on diabetes treatment for a long time just to give them the glucometer at home and then provide the strips every six months.’ (MGP6, male, 32, 4)

The study participants suggested engaging multiprofessional teams and the active involvement of individuals and their families to improve the control of diabetes. They were of the view that reinforcing community health workers’ participation, involving dieticians, and family support in providing care to patients with diabetes could improve the level of control of the disease:

‘Stakeholder’s involvement, for instance … you can call the community health care worker to go in and find out what is happening. So, after finding out what is happening to that, the family then is the one who can come and give you the report … so that you also involve the social worker. So, it will assist if the involvement of other stakeholders to take their part in the treatment of the diabetic clients.’ (NN1, female, 53, 25)

The family environment, including food preferences, could impact dietary modifications for a diabetic patient, according to the study participants. It becomes easier to modify the nutritional habits of a person living with diabetes if the family was involved in meal planning. In addition, it was suggested that community awareness campaigns through the media, and the use of health promoters should be intensified:

‘We need to educate patients; we need to educate the community, because sometimes even the family when they are cooking for the diabetic patients, they don’t have a special meal for that diabetic patient. They are just cooking, just like for everyone. So, it is just a basic thing, the lifestyle modification educating the patients and educating the family members.’ (MN3, male, 49, 22)

‘To make use of other stakeholders like the partners [*NGOs*] that can educate people about diabetes and also to make use of the health promoters they should have topics on diabetes each time they go out into the community.’ (FGP)

The study participants believed that setting aside an area for patients with chronic illnesses and designating staff for such patients could improve diabetes control, as the healthcare workers were more likely to focus discussions on diabetes at such designated areas:

‘If we can have the specialised chronic area, so that means when you do the education they are concentrating because they know that you are talking to them. Also, they will be open to you because if it is a whole hall, not everybody is interested in what you are saying. Even so, those patients who want to ask a question will be embarrassed because others will be laughing. So, it’s important to have a designated area for chronic clients.’ (NN3, female, 54, 25)

To address issues of limited resources, they suggested providing quality equipment, making diabetes management protocols available, training specialist diabetes nurses, and ensuring regular supply of medication. The focus group also indicated the need for a specific evidence-based guideline for diabetes management:

‘If they could address the issue of inferior quality of the test, the glucometer machine, because they keep on getting faulty, now, and again we are taking a new machine. Then also if the system could address the non-availability of drugs. And, if there could be protocols, specifically for diabetes for the professionals to access them at any given time when there’s a need.’ (NN9, female, 46, 22)

‘If government maybe would employ more nurses that are trained, then it could be better.’ (NN10, female, 29, 3)

‘So, if we could have a standardised protocol on how to manage diabetes to be in the Department of Health website or to have guidelines specifically for diabetic management of patients, I understand there are the guidelines for like the electronic EDL but also there should be one that is specifically for diabetes so that one can zoom in and get the information that she wants.’ (FGP)

The study participants emphasised the need to increase the staff members’ knowledge through in-service training and update courses:

‘What we can do, we must be well informed with the latest guidelines, and we should be taught about them; we must be orientated. We must go for workshops; we must go for in-service training so that our knowledge should be always up to date.’ (NN2, male, 39, 10)

## Discussion

This study highlighted primary health care workers’ views on the control of T2DM. Study participants perceived diabetes control had improved because they, recently, had not seen diabetic complications in patients at the clinics. The perceived improvements were attributed to the presence of medical doctors and professional nurses at the selected CHCs. These additions to the PHC teams enhanced the earlier identification of signs of complications and prompted earlier interventions by healthcare workers, according to study participants.

Similar findings of positive glycaemic outcomes and improved health status with intensive diabetes management was reported in a systematic review.^26^ While this study found the presence of medical doctors and professional nurses responsible for their perceived improvements in the control of diabetes, Murphy et al.^26^ found, in addition, that patient-oriented interventions such as adequate continuity of care; professional interventions such as proper disease management programmes; and financial interventions such as the provision of free medications, also contributed to improved control of diabetes.

We are, however, cautious in interpreting these views to be reflective of control of diabetes at the CHCs, because objective measures of diabetes control were not explored in our study. Also, the rates of screening for diabetes complications were low, as reported from a study conducted in a similar setting.^27^ Furthermore, patients with complications from diabetes may have presented to higher levels of care and not to the primary healthcare facilities in the first instance.

Despite perceiving an improved control of diabetes, study participants reported that control of the disease was still poor, and they identified some factors hindering the optimal control of diabetes in patients attending the CHCs.

The healthcare workers perceived that poor adherence to an ideal diabetic diet contributed to poor glycaemic control in patients with diabetes. The poor dietary adherence was ascribed to the patients’ poor socio-economic background. The drainage areas of the clinics in our study have high poverty rates,^23^ and poor socio-economic conditions constitute an important social determinant of health linked with poor dietary adherence.^28,29^ Hence, the perception of the study participants linking poor dietary adherence with poor glycaemic control is similar to findings by Romakin and colleagues in Fiji,^30^ and by Afroz and colleagues in Bangladesh.^31^

The study participants reported on the issue of diabetic patients’ non-commitment to scheduled clinic appointments as presenting an increased risk for poor control of the disease, and this is consistent with results from other studies.^30,31^ When a patient with diabetes misses scheduled clinic appointments, a string of missed opportunities ensues, leading to adverse disease outcomes in the long term. Some of the reasons given for missed scheduled clinic appointments include patients’ lack of transport money to the clinics, lengthy distances to the health facilities, fear of contracting COVID-19, and medication unavailability.

In addition to not honouring scheduled clinic appointments, the healthcare workers also perceived that patients’ low adherence to treatment also contributed to poor control of diabetes, and this had been reported previously.^30,32^ Our study participants believed that low treatment adherence was driven by patients’ fear of injections and poor treatment collection rates, again because of lack of transport fare to the clinic.

Study participants expressed that poor control of diabetes was linked to a poor understanding of the condition and that it was essential to enlighten patients with the disease through diabetes education, and this aligns with findings from previous studies.^32,33,34,35,36^ It is necessary, therefore, for healthcare workers to intensify their efforts in educating patients with diabetes on the nature of the disease and counselling the patients to enable them to become active participants in their own care. Yazdani et al., also advocated for prioritisation of strategies to overcome common barriers to achieving adequate education of patients.^35^

The study participants perceived that patients’ lack of personal blood testing equipment also contributed to poor control of diabetes. Similar findings were identified in Ethiopia^37^ and Mexico.^38^ In the literature, the practice of Self-Monitoring of Blood Glucose (SMBG) is promoted in patients with type 1 diabetes and in patients with insulin-treated type 2 diabetes.^39,40^ However, for patients with type 2 diabetes, SMBG seems to lead to slightly better glycaemic control only in the short term (less than 1 year).^39^ Therefore, the provision of personal glucometers to patients with type 2 diabetes, for SMBG, in the public healthcare sector, will have debatable long-term benefits in terms of glycaemic control.

This study also highlighted the effects of lack of support from family members and medical support services on the control of diabetes. A lack of a stable support system – loneliness in the elderly for example —, and lack of community health workers who regularly visit patients in their homes, negatively impacted on optimal glycaemic control, similar to findings by Whittemore et al.^38^

Accordingly, the services of multidisciplinary teams comprising community health workers, health promoters, social workers, dieticians, and home support, were suggested to be engaged to improve the control of diabetes. This opinion was also expressed by healthcare workers in the United States of America (US),^41^ who in addition, stated the need to encourage family members and co-workers to support patients living with diabetes. This support could be in the form of assisting the patients to overcome denial and fear of diabetes and its complications, implementing flexible work schedules where possible, reducing or eliminating competing responsibilities, and assisting with meal planning in a patient’s cultural context.

Physical infrastructure, materials, and human resources are required for the active and effective care of patients with diabetes at the PHC level.^42^ Our study highlighted that high patient volumes, long waiting times, limited resources to manage patients with diabetes, and delayed service provision, were associated with poor control of diabetes at the CHCs. These findings align with published literature in which similar factors were observed as barriers to achieving optimal glycaemic control.^38,40,42,43^

Participants in our study advocated for the creation of a separate area to manage patients with chronic diseases at CHCs. While the intention behind this suggestion may be to promote homogeneity in messaging, and to attain uniformity in management approach to such patients, this may lead to unintentional disclosure of health status on behalf of patients.

While findings from our study align with those in the literature, we note our study participants’ minimal emphasis on their role in educating patients and practising of patient-centred care, especially the important attribute of empathy required of healthcare workers for the management of diabetes. Improved staff empathy is vital in improving the bonding, and, in essence, the working alliance between patients and the health care providers for optimal management of the disease.^41^

### Limitations

This is a qualitative study with few participants, conducted in only two primary health care facilities, but the findings are likely to be transferable to similar settings in the health district and the province. The interviews were conducted in English language; however, it is unclear whether the use of the local predominant language could have enriched the responses in both individual and FGDs. Dieticians were not employed at the study sites during the study period. As such, the viewpoints of dieticians on diabetes, its management and way forward on improving glycaemic control could not be ascertained. Notwithstanding these limitations, the findings have highlighted important patients- and health system-related factors related to poor glycaemic control. In addition, the healthcare workers call for further training in diabetes management at the primary health care level in the country.

### Implications

There are some implications from our study. Poor socio-economic conditions continue to present several barriers to the adequate management of diabetes. It was also made apparent that patients living with diabetes may not have obtained the necessary education and counselling on the disease, in the context where our study was carried out. The role of the health care workers in the management of the disease, especially in demonstrating sufficient empathy with patients treated for diabetes should be emphasised. Primary health care workers involved with the management of diabetes should be regularly capacitated through update courses. Such courses also could emphasise the importance of improved attitudes of healthcare workers towards patients, while increasing the healthcare workers’ knowledge and skills.

## Conclusions

This study reported that healthcare professionals were of the perception that there was an improvement in the level of control of T2DM among patients at the CHCs. However, they also acknowledged the need for further improvements in the control of diabetes at the CHCs. They highlighted several patient and health system-related reasons for the poor management of diabetes at the primary health care level. These include poor dietary adherence, low socio-economic status, inadequate patient education and poor support systems, insufficient health infrastructure and healthcare professionals, inconsistent drug availability and low treatment adherence, long waiting times, and delayed service provision. The policymakers, healthcare providers, diabetic patients and community all have a part to play in ensuring optimum disease control.
